# Can biomedical and traditional health care providers work together? Zambian practitioners' experiences and attitudes towards collaboration in relation to STIs and HIV/AIDS care: a cross-sectional study

**DOI:** 10.1186/1478-4491-4-16

**Published:** 2006-07-17

**Authors:** Berthollet Bwira Kaboru, Torkel Falkenberg, Phillimon Ndubani, Bengt Höjer, Rodwell Vongo, Ruairi Brugha, Elisabeth Faxelid

**Affiliations:** 1 Division of International Health (IHCAR), Department of Public Health Sciences, Karolinska Institutet, Stockholm, Sweden; 2 Centre for Studies of Complementary Medicine, Division of International Health (IHCAR), Department of Public Health Sciences and Division of Nursing, Department of Neurobiology, Caring Sciences and Society, Karolinska Institutet, Stockholm, Sweden; 3 Institute of Economic and Social Research (INESOR), University of Zambia, Lusaka, Zambia; 4 Dalarna University College, Falun, Sweden; 5 Traditional Health Practitioners Association of Zambia (THPAZ), Lusaka, Zambia; 6 London School of Hygiene and Tropical Medicine (LSHTM), London, UK; 7 Division of Reproductive and Perinatal Care, Department of Women and Child Health, Karolinska Institutet, Stockholm, Sweden

## Abstract

**Background:**

The World Health Organization's *World health report 2006: Working together for health *underscores the importance of human resources for health. The shortage of trained health professionals is among the main obstacles to strengthening low-income countries' health systems and to scaling up HIV/AIDS control efforts. Traditional health practitioners are increasingly depicted as key resources to HIV/AIDS prevention and care. An appropriate and effective response to the HIV/AIDS crisis requires reconsideration of the collaboration between traditional and biomedical health providers (THPs and BHPs).

The aim of this paper is to explore biomedical and traditional health practitioners' experiences of and attitudes towards collaboration and to identify obstacles and potential opportunities for them to collaborate regarding care for patients with sexually transmitted infections (STIs) and HIV/AIDS.

**Methods:**

We conducted a cross-sectional study in two Zambian urban sites, using structured questionnaires. We interviewed 152 biomedical health practitioners (BHPs) and 144 traditional health practitioners (THPs) who reported attending to patients with STIs and HIV/AIDS.

**Results:**

The study showed a very low level of experience of collaboration, predominated by BHPs training THPs (mostly traditional birth attendants) on issues of safe delivery. Intersectoral contacts addressing STIs and HIV/AIDS care issues were less common. However, both groups of providers overwhelmingly acknowledged the potential role of THPs in the fight against HIV/AIDS. Obstacles to collaboration were identified at the policy level in terms of legislation and logistics. Lack of trust in THPs by individual BHPs was also found to inhibit collaboration. Nevertheless, as many as 40% of BHPs expressed an interest in working more closely with THPs.

**Conclusion:**

There is indication that practitioners from both sectors seem willing to strengthen collaboration with each other. However, there are missed opportunities. The lack of collaborative framework integrating maternal health with STIs and HIV/AIDS care is at odds with the needed comprehensive approach to HIV/AIDS control. Also, considering the current human resources crisis in Zambia, substantial policy commitment is called for to address the legislative obstacles and the stigma reported by THPs and to provide an adequate distribution of roles between all partners, including traditional health practitioners, in the struggle against HIV/AIDS.

## Background

The World Health Organization (WHO) has devoted the *World health report 2006: Working together for health *to human resources for health [1]. The shortage of trained human resources is among the most important obstacles to strengthening health systems in low-income countries [2,3] and to scaling up HIV/AIDS control initiatives. Sub-Saharan Africa, where around 25.8 million persons were reported living with HIV at the end of 2005, is affected most by this human resources crisis [4,5]. Providing prevention and care to the many millions at risk or already HIV-infected, most of whom live in remote rural areas and overcrowded urban environments, is a major challenge for African health care systems [4,6]. The complexities and sheer burden of caring for those affected by HIV/AIDS, with inadequate resources, is traumatizing modern health professionals [7].

Zambia is a country experiencing a serious shortage of health workforce due mainly to emigration and the low capacity of national medical and nursing schools, as well as deaths among health providers as a consequence of HIV/AIDS [8]. Efforts to control HIV/AIDS are thus jeopardized by an insufficient workforce.

Traditional health practitioners (THPs) are increasingly being depicted as crucial to scaling up care strategies for the millions of "hard-to-reach" people and communities affected by HIV/AIDS [6,9]. The major initiatives that have been launched to enhance human resources for health, such as the Rockefeller Joint Learning Initiative, have explicitly mentioned practitioners of traditional medicine, together with other community health workers, as being a crucial part of the health workforce to be considered [10]. According to UNAIDS, substantial proportions of the population use traditional health practitioners (THPs), who attend to around 60% of patients with sexually transmitted infections (STIs), including HIV/AIDS [11]. An appropriate and effective response to the HIV/AIDS crisis demands reconsideration of the interface between THPs and BHPs.

The relationships between modern and traditional health care providers in Africa have been characterized by tensions, denial and mutual repugnance since colonial times [12]. The literature provides a somewhat simplistic and unidimensional portrayal of modern and traditional health providers' attitudes towards each other and their willingness to collaborate. When this topic has been studied, THPs' views are often described, while BHPs' views are left out, and the attitudes of the two sectors are never compared. BHPs are generally portrayed as resisting collaboration, whereas THPs are presented as eager to collaborate with and learn from their biomedical counterparts [12-14].

Moreover, the advent of AIDS has in fact fuelled several types of collaboration between THPs and BHPs. Some types are less controversial and can easily be approved by both groups of providers (i.e. health education, training sessions, etc.), whereas other types are likely to raise fear and suspicion. This is the case with bioprospecting (the process of looking for valuable biochemical compounds from genetic resources, natural products or traditional knowledge), towards which many THPs might appear reluctant, for obvious reasons [15]. Thus far, there is a lack of systematic description, exploration and comparison of the experiences of and attitudes towards partnership and collaboration among a representative group of THPs and BHPs, in light of the AIDS pandemic.

The aim of this paper is to explore biomedical and traditional health practitioners' experiences of and attitudes towards collaboration and to identify obstacles and potential opportunities for them to collaborate with regard to care for patients with STIs and HIV/AIDS. By collaboration, we mean increased dialogue and communication between the two health sectors as well as various joint activities aimed at improving community health education and patients' care and support.

## Methods

### Context

This study was part of a broader project: "Bridging gaps between traditional and modern health care sectors – testing a model to improve quality of STI/HIV/AIDS care in sub-Saharan Africa". This 40-month project (2002–2006) involved four universities and two nongovernmental organizations (see Acknowledgement section). The ultimate objective of this project was to investigate whether dialogue, mutual respect, understanding and collaboration between traditional and modern health care providers would contribute to improving quality of care and uptake of STIs and HIV/AIDS services in Zambia and Uganda. The project comprised three phases: baseline, intervention and follow-up. This paper draws its data from the baseline phase, from which results were used to design the intervention.

### Study setting, design and sampling

A cross-sectional survey was conducted in two cities: Ndola (population 270 000) and Kabwe (population 175 000), in the Copperbelt and Central provinces of Zambia, respectively [16]. The study sites – southern zone of Ndola and northern zone of Kabwe – were selected on the basis of availability of health centres with STI/HIV services, especially voluntary counselling and testing (VCT) services as well as availability of THPs. The data were collected in May and June 2003.

The study population consisted of BHPs (nurses, midwives, physicians and laboratory and environmental health technicians) and THPs (herbalists, spiritualists, diviners and traditional birth attendants (TBAs)).

Twenty research assistants (RAs) were recruited to be data collectors (10 in each town). Ten of the RAs were THPs who interviewed fellow healers and ten were BHPs who interviewed fellow BHPs. The selected RAs underwent a one-week-long training to become familiar with the methods and the instruments. They were supervised on a daily basis by senior researchers from the collaborating institutions.

All six health centres in northern Kabwe and all seven in southern Ndola were included; we attempted to interview BHPs at those facilities. Respondents were interviewed individually, in a private area, at their respective health centres. Those on leave or off-duty were followed up and interviewed at home. All respondents who were reached agreed to be interviewed. The interviews with BHPs were conducted in English. Out of 172 BHPs eligible, 152 (80 in Kabwe and 72 in Ndola) were interviewed, while 20 (12%) could not be reached.

The district branches of the Traditional Health Practitioners Association of Zambia (THPAZ) provided lists of all THPs operating within the residential areas surrounding the selected health centres and from which the centres get their clients. In order to complement these lists and include non-THPAZ members, a systematic ambulatory mapping was conducted. This means that the RAs walked through these areas and asked residents about names and addresses of THPs they knew in the neighbourhood.

An updated list including names and residential addresses of the THPs was thus established, irrespective of their membership in THPAZ. The inclusion criterion was to select THPs who, according to their own statements, attended to patients with STIs and HIV/AIDS. All identified THPs consented to participate in the study. The total number of THPs interviewed was 144 (81 in Kabwe and 63 in Ndola). The THPs were interviewed in Bemba, the local language in both study areas.

The instruments used for data collection were structured questionnaires with open-ended and closed-ended questions. Most questions were the same for both groups. The questions in the instrument for THPs contained parallel translations in Bemba, as the interviews were conducted in that language.

### Analysis

The data were entered in EpiData version 3.0 (Epidata Association, Odense, Denmark, ), and analysed using STATA 8 (Stata Corporation, Texas, United States of America). Frequencies of responses were calculated and, for binary responses, the Pearson chi-square test (χ^2^) was applied to test the statistical difference between proportions (95% two-tailed significance). The analysis was done per group of providers rather than in terms of specific categories of THPs or of BHPs.

## Ethical issues

Approval for the study was obtained from the ethics committees at Karolinska Institutet (Sweden), the University of Zambia (Zambia) and the London School of Hygiene and Tropical Medicine (United Kingdom).

## Results

### Background characteristics of the respondents

The average age of THPs was 48 years (range 26–90), with 75 (52%) women and 69 (48%) men. Overall, 116 (81%) of the THPs were literate. The BHPs were younger – average age 39 years (range 19–56) – and included 132 (87%) women and 20 (13%) men. Among BHPs, 128 (84%) were nurses and midwives, 7 (5%) were clinical officers, 2 (1%) were medical officers, and the remaining 15 (10%) were environment health specialists, laboratory technicians and other auxiliary workers.

### Experience of intersectoral collaboration

Respondents were asked if they had had any type of collaboration with counterparts in the other health sector in the previous six months. Thirty-seven BHPs (24%) affirmed that they had, compared to 19 (13%) of the THPs (p = 0.015). Twenty-seven BHPs (69%) reported that the collaboration consisted of training of TBAs in conducting safer deliveries. For THPs, most (24%) reported having been trained by BHPs on HIV/AIDS matters (Table 1).

**Table 1:** Types of collaboration as stated by providers who affirmed that they were involved in collaboration

**Responses from BHPs (n = 37)**		**Responses from THPs (n = 19)**	
Types of collaboration	Frequencies (%)	Types of collaboration	Frequencies (%)

Training THPs on deliveries	27 (69.2)	Training by BHPs on HIV/AIDS matters	5 (23.8)
Health education on STI/HIV/AIDS	4 (10.3)	Cross-referrals for care	4 (19.0)
Advised THPs on hygiene	4 (10.3)	Joint STI/HIV health education	4 (19.0)
Visited/observed THPs' work	2 (5.1)	Joint health education activities	3 (14.3)
Training THPs on counselling mothers on HIV	1 (2.6)	We referred patients for lab tests	2 (9.5)
Training THPs on diet	1 (2.6)	Trained by BHPs on deliveries	2 (9.5)
Missing	2 (5.1)	Other meetings	1 (4.8)

Because multiple responses were given, the sum of responses may be >100%

When asked about their own referral practices, six BHPs (4%) reported having referred patients to THPs and 76 THPs (53%) reported having referred patients to BHPs (non-response from one THP). The providers who reported not having referred to the other sector enumerated the reasons for not doing so, as shown in Table 2. The most common response among BHPs was the presence of rules against referrals, followed by lack of trust in THPs. The THPs cited the lack of formal collaborative mechanisms, their own ability to treat and resistance from BHPs as reasons for not having referred to the other sector.

**Table 2:** Reasons for not referring among providers who had not referred patients to the opposite sector

**Responses from BHPs(n = 146)**		**Responses from THPs (n = 67)**	
Perceived reasons for not referring to THPs	Frequencies (%)	Perceived reasons for not referring to BHPs	Frequencies (%)

Rules (referral guidelines)	34 (23.3)	No collaboration mechanism	26 (38.8)
Lack of trust in THPs	28 (19.2)	I can treat	18 (26.9)
No need to refer	18 (12.3)	BHPs don't accept us & our patients	18 (26.9)
BHPs are better	17 (11.6)	I have a few patients	5 (7.5)
BHPs fear losing patients	16 (10.7)	BHPs don't refer to us	4 (5.9)
Lack of belief in THPs	15 (10.3)	Other	7 (10.4)
Lack of knowledge about THPs' practice	11 (7.5)		
Referral only from THPs-BHPs	10 (6.8)		
Other	1 (0.7)		

Because multiple responses were given, the sum of responses may be >100%

Twenty-eight THPs (19%) reported that they had received patients referred to them by BHPs (non-response from one THP) and 38 (25%) of the BHPs reported that they had received patients referred from THPs (p = 0.214). The respondents who did not report any reception of referral were asked about their perceptions of the reasons why their counterparts did not refer patients to them (Table 3). Most BHPs stated that THPs could treat patients and did not need to refer and that THPs feared losing patients' trust. Close to half of THPs cited BHPs' dislike of healers as reasons for BHPs' not referring to them, with lack of collaborative mechanisms being an important reason.

**Table 3:** Reasons for the opposite sector not referring patients (as perceived by providers who have not received referred patients)

**Responses from BHPs (n = 114)**		**Responses from THPs (n = 115)**	
Perceived reasons for THPs' not referring patients to BHPs	Frequencies (%)	Perceived reasons for BHPs' not referring patients to THPs	Frequencies (%)

THPs can treat	39 (34.1)	BHPs dislike us	49 (42.6)
THPs fear losing patients' trust	19 (16.7)	No collaboration mechanisms	37 (32.2)
No collaboration mechanisms	15 (13.2)	BHPs don't know us	21 (18.3)
THPs fear losing money	13 (11.4)	Not sure	3 (2.6)
No referral system	5 (4.4)	BHPs can treat	2 (1.7)
No trust in modern medicine	5 (4.4)	Other	14 (12.2)
THPs fear of critics	3 (2.6)		
Don't know	3 (2.6)		
Other	8 (7.0)		
Missing	4 (3.5)		

Because multiple responses were given, the sum of responses may be >100%

### Attitudes towards learning from each other

Providers' attitudes towards learning from each other were generally positive, although more so on the THPs' side: 117 BHPs (77%) and 140 THPs (97%) believed that BHPs could learn from THPs. Likewise, 147 BHPs (97%) and 129 THPs (90%) believed THPs could learn from BHPs.

### THPs' role in the fight against HIV/AIDS

Both groups, but more THPs, believed that THPs had a role to play in the fight against HIV/AIDS, including 126 BHPs (83%) and 136 THPs (97%). Possible roles for THPs were explored with both sets of respondents who believed such roles exist. Health education (including HIV prevention) and treatment of opportunistic infections and/or STIs were the predominant roles for THPs, as suggested by BHPs and THPs (Table 4).

**Table 4:** Potential roles of THPs in HIV/AIDS struggle (by providers who believed that such roles existed)

**Responses from BHPs(n = 126)**		**Responses from THPs (n = 136)**	
Views about the roles of THPs	Frequencies (%)	Views about the roles of THPs	Frequencies (%)

Health education	95 (75.4)	Treatment of opportunistic infections and STIs	72 (52.9)
Treatment of opportunistic infections and STIs	31 (24.6)	Health education	42 (30.9)
Condom distribution	15 (11.9)	Participation in research activities	31 (22.8)
Use of sterile tools	10 (7.9)	Condom distribution	7 (5.1)
Identify and refer cases	9 (7.1)	Counselling	5 (3.7)
Counselling	8 (6.3)	Spiritual healing for AIDS	3 (2.2)
Other	12 (9.5)	Use of sterile tools	2 (2.2)
		Other	7 (5.1)

Because multiple responses were given, the sum of responses

### Interest in closer collaboration

Sixty-one BHPs (40%) and 139 THPs (97%) expressed an interest in working together with the other sector. As to the ways to do this, the providers who stated that they were willing to collaborate gave a number of suggestions (Table 5). Meetings and cross-referrals were the most common options mentioned, respectively, by BHPs and THPs.

**Table 5:** Suggested ways of collaboration among providers who expressed a wish to collaborate

Responses from BHPs(n = 61)		Responses from THPs(n = 139)	
How to collaborate	Frequencies (%)	How to collaborate	Frequencies (%)

Meetings/Workshops/Cross-visits	23 (37.7)	Cross-referrals	41 (29.4)
Learning about traditional medicine	13 (21.3)	Work together	35 (25.2)
Work together	6 (9.8)	Collaboration/respect/recognition	26 (18.7)
THPs refer to BMs	6 (9.8)	Meetings/Workshops/Cross-visits	15 (10.7)
Visit THPs	5 (8.2)	Learning about biomedicine	14 (10.0)
Refer patients to THPs	4 (6.6)	Joint research programmes	11 (7.9)
Train THPs	3 (4.9)	Other	9 (6.5)
Joint research programmes	2 (3.7)		
Cross-referrals	2 (3.7)		
Lobbying together for policy change	2 (3.7)		
Other	4 (6.6)		

Because multiple responses were given, the sum of responses may be >100%

## Discussion

This study showed that, despite advocacy by the WHO [17] and UNAIDS [11], collaboration between BHPs and THPs in care and prevention of STIs and HIV/AIDS remains weak in Zambia. Only 24 % of BHPs and 13 % of THPs reported contacts with the other sector within the previous six months. Additionally, the types of past collaboration consisted mainly of the traditional approach of BHPs training THPs, rather than the two groups learning together and from each other. This "one-way" training is at variance with the "two-way education" identified in a previous study as a prerequisite for successful collaboration [18]. Moreover, most BHPs reported that they had trained THPs on safe delivery, whereas THPs generally reported having received training from BHPs on HIV/AIDS. This discrepancy points to an absence of a comprehensive approach in which STIs and HIV/AIDS and maternal health are integrated in the intersectoral collaborative efforts.

Although referrals should not be seen as the ideal yardstick of collaboration or of willingness to collaborate, the mere existence of referrals implies a belief that the other provider will offer something beneficial to the patient. Reported views on referrals reflect the nature of attitudes of providers towards each other. Thus, perceptions of what hinders the opposite sector from referring were informative. Interestingly, one third of BHPs believed that THPs could treat medical conditions, suggesting a positive view of THPs' abilities. Fear of losing clientele and patients' trust were cited by BHPs as reasons for THPs' not referring. This fear has often been seen as a hindrance to collaboration [14]. In addition, THPs' perceived reasons for BHPs' not referring to them suggested frustration (e.g. beliefs that BHPs dislike them, do not know them, do not accept them and their patients). Although the responses came from THPs themselves and were not expressly confirmed by BHPs, they reflect the stigma that THPs experience. THPs' image needs, perhaps, to be improved in the public health discourse if they are to be seen as fully-fledged and respect-worthy partners.

Both groups of health care providers believed in the value of the other sector and many were favourable to collaboration, suggesting it is possible if well designed. Indeed, sizable proportions of BHPs and THPs believed THPs had a role to play in HIV/AIDS control and that the two groups could learn from each other; they also expressed interest in working more closely. As to the roles of THPs, health education and treatment of opportunistic infections and STIs were predominantly mentioned by both groups of providers. There are indications that traditional medicine and its practitioners have an important role in the management of some opportunistic infections [17,19]. Although substantiation of this hypothesis was not an objective of this study, the opportunity to learn about traditional medicine was the second most common form of collaboration recommended by BHPs, and was another important indication of their positive attitudes towards and interest in traditional practices.

Homsy et al. have suggested THPs as a potential entry point and resource for the provision of VCT, prevention of mother-to-child HIV transmission and support to antiretroviral treatment provision [6]. Furthermore, according to a recent study from Tanzania, THPs are adapting their interventions (by combining herbal medication and counselling), as a response to HIV/AIDS. The HIV/AIDS crisis has thus been described as a catalyst bringing healers, policy-makers and biomedical workers together [20]. This remains to be seen in Zambia. A recent study commissioned by the United States Agency for International Development (USAID) to identify possible solutions to enhance human resources for HIV/AIDS care in Zambia has suggested a number of delegations of tasks in the delivery of care: from physicians to clinical officers and nurses and from lab technicians to other lower-level health workers. The report, however, made no mention of the available resources in the traditional sector [8].

Health education, which is recognized by both groups of providers as a potentially important role for THPs, is an area where collaboration is needed in Zambia. A recent sexual behaviour survey found a very low level of involvement of both groups of providers in prevention activities at the community level [21]. Collaboration could strengthen both sectors by developing synergies between them, by sustaining cultural sensitivity of AIDS programmes and by expanding their coverage [22].

As to mechanisms for launching and developing collaboration at the primary service delivery levels, THPs favoured concrete and collaborative actions from the start, especially bidirectional referrals, indicating a wish for practical (rather than rhetorical) recognition. Meetings were the most common collaborative mechanism suggested by BHPs, indicating that they favoured a more cautious and gradual approach. The biggest policy obstacle to overcome seemed to be that of referrals from BHPs to THPs. It appears that BHPs do not feel protected in making overt referrals to THPs.

A likely and important consequence of this may be that referrals take place in an informal and covert way, with neither patients nor providers wishing to publicize the practice. Other constraints related to logistical issues such as transport difficulties have been reported to obstruct intersectoral cooperation and the provision of health services elsewhere in Zambia [23,24]. However, even when regulation allows referrals, there is a need to clarify and agree on the conditions for which referrals should be made.

At the global level, despite the acknowledgement of the potential role of THPs in UNAIDS/WHO publications over the last five years [11,17,25], more recent WHO plans for achieving "3 by 5" (putting three million people on antiretroviral treatment by end of 2005) made little or no mention of a role for the traditional sector [4]. It can therefore be argued that failure to mobilize these resources might have contributed to the poor results of the "3 by 5" strategy.

There is an urgent need to involve BHPs, THPs, patients and other stakeholders, preferably by means of participative research methodologies, to explore ways of setting up effective strategies to tackle key HIV/AIDS care issues through improvement of intersectoral collaboration. The health benefits of collaboration to patients and its impact on the overall health system are also aspects that will need particular research attention. Further research is called for in order to capitalize on the increasing interest in traditional medicine on the part of modern practitioners [24,26,27]. This understanding might help identify contextualized collaborative strategies. Carefully managed and evaluated pilot interventions are needed. These should be agreed to by traditional and modern practitioners, but also supported from outside.

The methodology used in this study had strengths and limitations. One strength was the sampling of all eligible participants in the study settings, as this minimized sampling bias. Biases were possible, by using THPs or BHPs as research assistants to interview their colleagues, where respondents might have wanted not to be seen as deviant and, thus, might have tried to conform to commonly accepted views in their profession. The advantages in terms of access, mutual understanding and optimal response rates were the rationales for their choice as research assistants; training was relied upon to minimize the risks of such biases.

We also recognize that THPs who reported not attending to patients with STIs and HIV/AIDS might have done so unknowingly and would therefore have been interviewed, as they also might provide valuable suggestions regarding collaboration. However, we opted for their exclusion because we sought the opinions of those with actual experience of treating STIs and HIV/AIDS so that their accounts would contribute to the design of an intervention.

Another limitation is that we did not divide THPs into different categories. This attempt was abandoned as we realized that it could not be done in a straightforward manner, since many Zambian THPs actually had many specialities at the same time.

## Conclusion

This study confirms a low level of experience of overt collaboration between the two groups of providers, but recognition by both sectors of the role of THPs in the fight against HIV/AIDS. This study provides evidence that the current policy environment (legislation), together with BHPs' lack of trust in THPs, are major obstacles that justify their fear of not openly referring patients to THPs; and that logistical constraints are an additional impediment to intersectoral collaboration. Also, the stigmatization expressed by THPs and the lack of recognition are issues that need to be addressed.

A key observation is that the current, limited interactions are in line with the old paternalistic approach of one group being taught by another, and favour only TBAs, and thus mainly maternal health issues. This approach is inappropriate in a context of needed comprehensive HIV care, given the scale of the AIDS epidemic and the shortage of human resources in Zambia. The challenge is to define a broader framework that recognizes all relevant actors in HIV/AIDS control as useful partners and that allows for a distribution of roles among them.

## Competing interests

The author(s) declare that they have no competing interests.

## Authors' contributions

BBK was instrumental in the analysis and interpretation of the data, in the drafting of the manuscript and in the discussion of policy relevance of the findings of the study. TF was involved in the study design, the interpretation of the data and in the discussion of the policy relevance of the findings. PN provided a crucial contribution to the data collection process, to the interpretation of the findings and the drafting of the article. BH participated in the study design and the interpretation of the data. RV actively participated in the data collection process and reviewed the policy relevance of the findings. RB brought a crucial contribution to the data interpretation, the drafting of the manuscript and to the discussion on policy relevance of the study. EF coordinated the process from the study design to the drafting of the article and revisited critically the manuscript to ensure required intellectual quality. The other members of the Bridging Gaps Project's Research Team participated in the collection of data.
